# A Solvent Exchange Induced Robust Wet Adhesive Hydrogels to Treat Solid Tumor Through Synchronous Ethanol Ablation and Chemotherapy

**DOI:** 10.1002/advs.202309760

**Published:** 2024-04-06

**Authors:** Yanlv Chen, Meng Yu, Menghui Liu, Yang Sun, Chengxian Ling, Mingyu Yu, Wenwen Zhang, Wenkai Zhang, Xin Peng

**Affiliations:** ^1^ Guangdong Provincial Engineering Research Center of Molecular Imaging The Fifth Affiliated Hospital Sun Yat‐sen University Zhuhai 519000 China; ^2^ Guangdong‐Hong Kong‐Macao University Joint Laboratory of Interventional Medicine The Fifth Affiliated Hospital Sun Yat‐sen University Zhuhai 519000 China; ^3^ Department of Neonatology The Fifth Affiliated Hospital Sun Yat‐sen University Zhuhai 519000 China; ^4^ Department of Interventional Medicine The Fifth Affiliated Hospital Sun Yat‐sen University Zhuhai 519000 China; ^5^ Department of Orthopedics The Fifth Affiliated Hospital Sun Yat‐sen University Zhuhai 519000 China

**Keywords:** adhesive hydrogel, antitumor, chemotherapy, ethanol ablation, solvent exchange

## Abstract

The treatment of tumors in developing countries, especially those with poor medical conditions, remains a significant challenge. Herein, a novel solvent‐exchange strategy to prepare adhesive hydrogels for the concurrent treatment of tumors through synchronous ethanol ablation and local chemotherapy is reported. First, a poly (gallic acid‐lipoic acid) (PGL) ethanol gel is prepared that can undergo solvent exchange with water to form a hydrogel in situ. PGL ethanol gel deposited on the wet tissue can form a hydrogel in situ to effectively repel interfacial water and establish a tight contact between the hydrogel and tissue. Additionally, the functional groups between the hydrogels and tissues can form covalent and non‐covalent bonds, resulting in robust adhesion. Furthermore, this PGL ethanol gel demonstrates exceptional capacity to effectively load antitumor drugs, allowing for controlled and sustained release of the drugs locally and sustainably both in vitro and in vivo. In addition, the PGL ethanol gel can combine ethanol ablation and local chemotherapy to enhance the antitumor efficacy in vitro and in vivo. The PGL ethanol gel‐derived hydrogel shows robust wet bioadhesion, drug loading, sustained release, good biocompatibility and biodegradability, easy preparation and usage, and cost‐effectiveness, which make it a promising bioadhesive for diverse biomedical applications.

## Graphical abstract

After undergoing solvent exchange with water, an injectable PGL ethanol gel can transform into a robust wet adhesive hydrogel, which can be used to treat tumors through synchronous ethanol ablation and local chemotherapy.

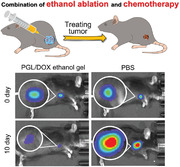

## 1 Introduction

In recent decades, in situ tumor ablation technologies have attracted increasing attention as minimally invasive surgeries for the local treatment of solid tumors. These techniques, including ethanol ablation,^[^ ^1^ ^]^ radiofrequency ablation,^[^ ^2^ ^]^ microwave ablation,^[^ ^3^ ^]^ high‐intensity focused ultrasound,^[^ ^4^ ^]^ and cryoablation,^[^ ^5^ ^]^ offer direct mechanisms for tumor cell destruction. In particular, ethanol ablation has garnered substantial attention due to its suitability for use in resource‐constrained settings, such as developing countries, where advanced medical resources may be limited. Ethanol ablation involves the direct injection of ethanol into malignant tissue to induce necrosis through processes like cell dehydration, protein denaturation, and microvascular thrombosis. Its key advantages include its ultra‐low‐cost (around $110 per treatment) no special equipment requirement, high portability, and the ability to treat relatively large lesions of up to 5 cm in diameter.^[^ ^6^ ^]^ However, the presence of fiber tissues in the tumor poses a challenge when completely filling the tumor with ethanol. Moreover, when tumors have several lesions or are large, ethanol can easily be diluted or washed away due to increased blood supply. This may result in the need for a larger dose of ethanol, potentially leading to toxicity in surrounding normal tissues.^[^ ^7^ ^]^ These shortcomings highlight the difficulties of treating tumors with ethanol ablation alone.

Chemotherapy has long been a traditional strategy for tumor treatment, but is associated with significant side effects on normal tissues throughout the body.^[^ ^8^ ^]^ To mitigate these side effects and enhance the therapeutic outcome, targeted antitumor drug delivery has emerged as a promising strategy using injectable hydrogels. For example, Guo et al. locally delivered Silibinin to lung adenocarcinoma via an injectable and biodegradable hydrogel composed of pectin hydrazide and oxidized carboxymethyl cellulose. This method significantly enhanced the antitumor efficiency in vivo and greatly reduced the toxicity of Silibinin.^[^ ^9^ ^]^ Jokerst et al. developed an injectable supramolecular hydrogel that can slow and track the release of doxorubicin (DOX) via photoacoustic tomography. And this hydrogel can slow the rate of the murine intraperitoneal tumor growth by 72.2% more than the free DOX group.^[^ ^10^ ^]^ Chen et al. designed a cisplatin‐crosslinked hyaluronic acid nanogel for delivering DOX to treat osteosarcoma, which released cisplatin and DOX into the intracellular acidic environment achieving synergistic therapeutic effects.^[^ ^11^ ^]^ Santos et al. reported an injectable and self‐healing hydrogel for the local delivery of Mo_154_ and DOX, enabling synchronous chemo‐photothermal tumor therapy.^[^ ^12^ ^]^ Therefore, targeted delivery of antitumor drugs to tumors through injectable hydrogels is an effective strategy to treat tumors.

The introduction of a polymer from a good solvent (such as ethanol, dimethyl sulfoxide, or *N*, *N*‐dimethylformamide) to a poor solvent (like water), can trigger a transformation in the polymer chains, causing them to shift from a loose conformation to an aggregated state. This process involves the creation of intra‐ and inter‐polymer hydrogen bonds and hydrophobic aggregation via solvent exchange, resulting in the formation of a hydrogel in situ.^[^ ^13^ ^]^ As a result, a solvent exchange‐induced hydrogel has the potential to introduce ethanol into the malignant tissue, maintain the local concentration of ethanol, and facilitate the targeted delivery of antitumor drugs. However, to the best of our knowledge, no solvent exchange‐induced hydrogels have been applied to treat solid tumors using synchronous ethanol ablation and chemotherapy.

Gallic acid (GA) and lipoic acid (LA) are soluble in ethanol, but insoluble in water at room temperature. GA is derived from plant extracts and contains phenolic hydroxyl and carboxylic acid groups. Conversely, LA is an essential coenzyme found in animal mitochondria and contains 1,2‐dithiolanes and carboxylic acid groups.^[^ ^14^ ^]^ The 1,2‐dithiolanes present in LA serve a dual purpose. They can undergo thermal ring‐opening polymerization to form poly (lipoic acid) (PLA). Additionally, they can also undergo a thiyl radical–polyphenol Michael addition reaction with GA to form poly (gallic acid‐lipoic acid) (PGL).^[^ ^15^ ^]^ Upon contact with water, the phenolic hydroxyl and carboxylic acid groups in PGL form hydrogen bonds with each other. Simultaneously, the hydrophobic segment in PLA forms a hydrophobic domain. Therefore, GA and LA are the best choice of materials for preparing ethanol‐water exchange‐induced hydrogels.

**Figure 1:**
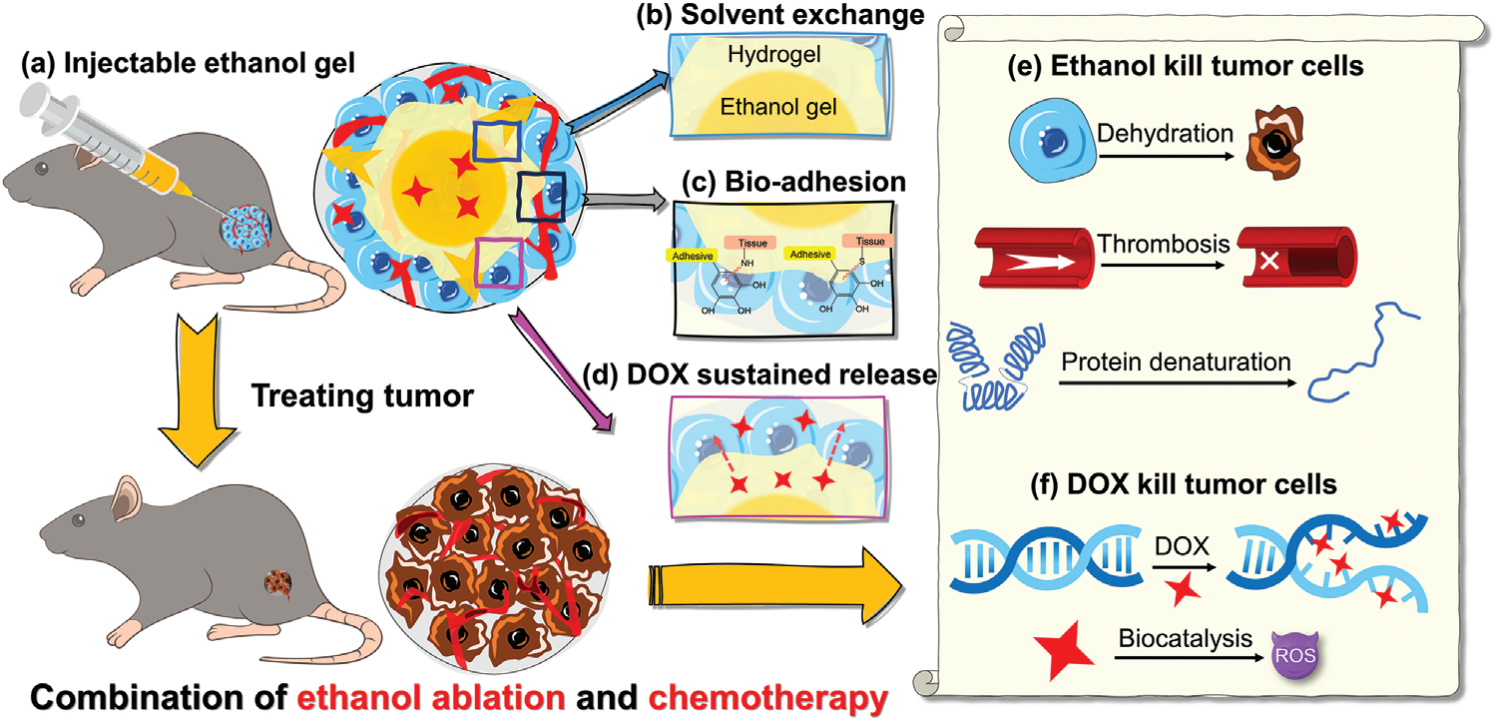
Schematic illustration of solvent exchange induced wet adhesive hydrogel for treating solid tumor through synchronous ethanol ablation and chemotherapy.

Herein, we report an injectable PGL ethanol gel that can undergo a solvent exchange to transform into an in situ formed hydrogel with robust wet bioadhesion. Furthermore, the introduction of DOX into the PGL ethanol gel can help treat subcutaneous Hepa 1–6 tumors through synchronous ethanol ablation and chemotherapy. First, based on the thiyl radical–polyphenol Michael addition reaction and physical interactions, an injectable PGL ethanol gel was synthesized via a one‐pot reaction. Upon contact with water, the PGL chains transformed from a loose conformation to an aggregated state. Consequently, the PGL polymer chains instantaneously formed intra‐ and inter‐polymer hydrogen bonds and hydrophobic aggregates, resulting in the formation of a PGL hydrogel in situ. The hydrophobic segment in the PLA hydrogel can then repel the residual interfacial water to form tight contact with the tissue. The functional groups in PGL can form covalent and non‐covalent bonds with the functional groups in the tissue, inducing robust adhesion between in situ formed PGL hydrogel and various tissues. Moreover, the PGL hydrogel exhibited good biocompatibility and biodegradability, both in vitro and in vivo. In addition, the adhesive PGL hydrogel formed in situ is capable of gradually releasing DOX at the target site. Finally, by leveraging the tumor‐killing properties of ethanol, which acts through cell dehydration, protein denaturation, and microvascular thrombosis, in combination with DOX, which kills tumors via DNA damage and the generation of reactive oxygen species (ROS), we have successfully demonstrated the effectiveness of PGL ethanol‐derived hydrogel in treating subcutaneous tumors in rat models through synchronous ethanol ablation and chemotherapy (**Figure** **1**).

## 2 Results and Discussion

### 2.1 Preparation and Properties of the PGL Ethanol Gel and PGL Hydrogel

**Figure 2:**
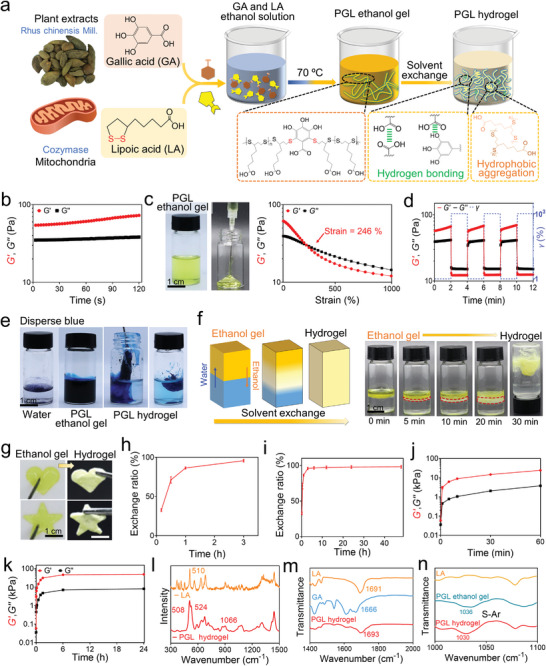
Preparation and properties of the PGL ethanol gel and PGL hydrogel. a) Schematic illustration of the preparation of PGL ethanol gel and PGL hydrogel. b) Time–sweep measurements of a PGL ethanol gel at 37 °C (frequency = 10 rad s^−1^ and *γ* = 1%). c) Photos of the PGL ethanol gel (left) and train–sweep measurements of a PGL ethanol gel at 37 °C (frequency = 10 rad s^−1^) (right). d) Repeated dynamic strain step testing (*γ* = 1% or 1000%, frequency = 10 rad s^−1^) of a PGL ethanol gel at 37 °C. e) Photos of the solvent exchange between PGL ethanol gel and water. f) Schematic illustration (left) and photos (right) of the change process from PGL ethanol gel to PGL hydrogel. g) Injection of PGL ethanol gel into molds for preparing hydrogels with various shapes. h,i) The ethanol‐water exchange ratio of PGL ethanol gel after being immersed in water. j,k) Change in *G′* and *G″* of the PGL hydrogels after being immersed in SGF. l) Raman spectra of the LA and dried PGL hydrogel. m,n) FTIR spectra of the LA, GA, PGL ethanol gel and dried hydrogel. LA: lipoic acid, GA: gallic acid, PGL: poly (gallic acid‐lipoic acid). Data are shown as the mean ± standard deviation (SD).

Ethanol, a key reagent for tumor ablation, was used as the solvent to dissolve GA and LA (**Figure** **2**a; Figure S1, Supporting Information). At 70 °C, the 1,2‐dithiolane of LA underwent thermal‐initiated ring‐opening polymerization to form PLA through dynamic disulfide exchange.^[^ ^15b^ ^]^ Subsequently, the PLA was able to react with GA through a thiyl radical–polyphenol Michael addition reaction, resulting in the synthesis of PGL. Furthermore, the storage modulus (*Gʹ*) of the obtained sample was higher than its loss modulus (*Gʹʹ*), indicating that a PGL ethanol gel can be acquired via a one‐pot reaction (Figure 2b). Due to the relatively weak interpolymer interactions of PGL in ethanol, the obtained PGL ethanol gel was injected through a 16 G syringe needle (Figure 2c; Video S1, Supporting Information). The strain sweep test revealed that both *G′* and *G″* of the PGL ethanol gel decreased with increased strain (*γ*), with *G″* eventually exceeding *G′* at *γ* = 246%, indicating favorable shear‐thinning properties (Figure 2c). To characterize the mechanical stability of the PGL ethanol gel after injection through a needle, we tested the recovery of the modulus during cyclic high‐low strain. These tests demonstrated that the ethanol gel rapidly transitioned from a solid‐like behavior (*G′* > *G″*) to a liquid‐like behavior (*G″* > *G′*) after the application of high strain (*γ* = 1000%) and subsequently recovered its solid‐like properties after the application of low strain (*γ* = 1%) (Figure 2d). Therefore, the results confirm that an injectable PGL ethanol gel can be readily obtained via a one‐pot reaction.

Upon contact with water, the PGL chains in ethanol changed from a loose conformation to an aggregated state in water. This transition prompted the PGL to instantaneously establish intra‐ and inter‐polymer hydrogen bonds and hydrophobic aggregates, which promoted the ethanol gel‐hydrogel transition and enhanced cohesion (Figure 2a). To observe the solvent‐exchange process, we added a dispersed blue dye, which is soluble in ethanol and insoluble in water, to the PGL ethanol gel. After getting in contact with the simulated physiological fluid (SPF), ethanol diffused out of the gel into the water, causing the water to infiltrate the polymer network. This resulted in the formation of a blue ethanol aqueous solution and a blue adhesive hydrogel (Figure 2e). Additionally, to observe the change from a PGL ethanol gel to a PGL hydrogel, we deposited a layer of PGL ethanol gel onto the SPF. Upon contact with water, the golden‐yellow transparent ethanol gel at the interface transitioned into a light‐yellow opaque hydrogel. Subsequently, the gradual solvent exchange between the ethanol gel and water induced an increase in the light‐yellow opaque area over time, until a uniform light‐yellow opaque hydrogel was formed (Figure 2f). In addition, by injecting PGL ethanol gel into molds of different shapes and immersing them in SPF, custom freestanding PGL hydrogels with corresponding shapes were obtained. This demonstrates the potential of the PGL hydrogel to fit irregularly shaped target sites (Figure 2g).

Next, we studied the ethanol‐water exchange process. As shown in Figure 2h, in the first 1 h, the ethanol‐water exchange was very fast, and 86.5 ± 1.3% ethanol was replaced by water. Then, the ethanol‐water exchange slowed down, and the exchange ratio reached 95.7 ± 1.8% at 3 h. Finally, the solvent exchange reached an equilibrium state, resulting in a 98.1 ± 1.9% exchange ratio at 48 h (Figure 2i). On the other hand, we examined the mechanical properties of PGL hydrogels using rheological tests. Upon contact with SPF, the *G′* of the sample sharply increased from 58.7 ± 5.5 to 3056 ± 513.6 Pa, indicating substantially enhanced cohesion from the ethanol gel to the hydrogel. Furthermore, the *G′* and *G″* of the PGL hydrogels significantly increased after being immersed in SPF for 24 h (Figure 2j,k). This can be attributed to the polymer forming stronger interactions with each other over time, inducing denser cross‐linking densities and higher *G′*.

Finally, we investigated the crosslinking mechanism of the PGL hydrogels. The Raman spectrum indicated that LA exhibited a disulfide bond (S─S) at 510 cm^−1^, while the PGL displayed two new peaks at 508 and 524 cm^−1^, highlighting the opening polymerization of LA. Meanwhile, a new peak (1066 cm^−1^) of PGL suggested the formation of a sulfur‐aromatic bond (S─Ar) between LA and GA, signifying that LA was grafted onto the benzene ring of GA through the Michael addition reaction (Figure 2l). Furthermore, the Fourier‐transform infrared (FTIR) spectra of LA, GA, and dried PGL hydrogels revealed that the carboxylic acid peaks at 1691 and 1666 cm^−1^ in LA and GA shifted to 1693 cm^−1^ in the PGL hydrogel. This shift indicated that the carboxylic acid group in LA formed hydrogen bonds with the carboxylic acid and phenolic hydroxyl groups in GA (Figure 2m). Additionally, the sulfur‐aromatic bonds peak at 1036 cm^−1^ in the PGL ethanol gel and shifted to 1030 cm^−1^ in the hydrogel, suggesting the hydrophobic interactions in the PGL hydrogel (Figure 2n). In general, GA and LA can react in ethanol to form a PGL ethanol gel. Subsequently, PGL can undergo inter‐ and intra‐polymer physical interactions (such as hydrogen bonding and hydrophobic aggregation) upon contact with water, resulting in the formation of PGL hydrogels.

### 2.2 Robust Wet Bio‐Adhesion of the PGL Ethanol Gel‐Derived Hydrogel

**Figure 3:**
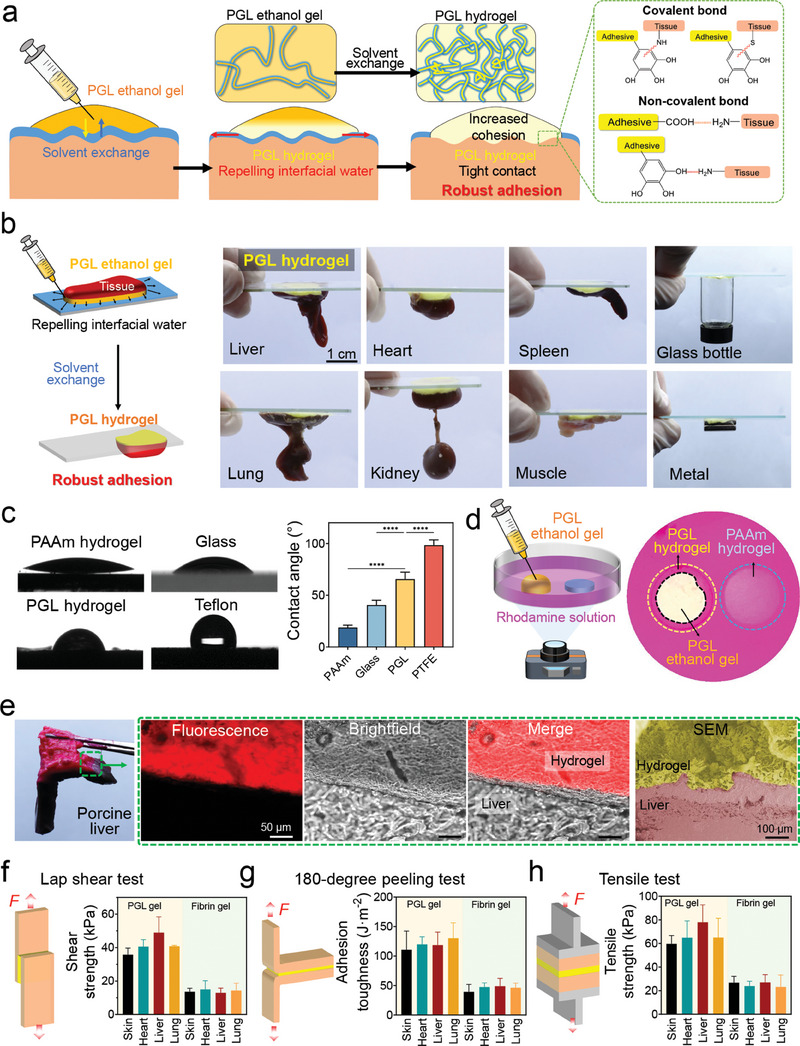
Robust wet bio‐adhesion of the PGL ethanol gel‐derived hydrogel. a) Schematic illustration of the adhesion of the PGL ethanol gel‐derived hydrogel on the wet tissue. b) Schematic illustration (left) and photos of the adhesion of the PGL ethanol gel‐derived hydrogel on various substrates including rat's liver, lung, heart, kidney, spleen, muscle, and glass bottle, metal. c) The contact angle of the water on various substrates, including the PAAm hydrogel, glass plate, PGL hydrogel, and Teflon plate. d) The water repulsion of the PGL ethanol gel. e) Microscopic and SEM photos of the cross‐section of the interface of the PGL hydrogel and porcine liver. f–h) Shear strength (f), adhesion toughness (g), and tensile strength (h) of the PGL ethanol gel‐derived hydrogel and commercial injectable adhesive fibrin gel on various tissues after immersing samples in PBS for 1h, including porcine skin, heart, liver, and lung. Statistical significance was calculated by Student's *t*‐test. ^****^ *p* < 0.0001. Data are shown as the mean ± SD.

We hypothesized that the wet adhesive mechanism of the PGL ethanol‐gel‐derived hydrogel was as follows: Upon injection onto wet tissues, the PGL ethanol gel instantly undergoes solvent exchange and transforms into a hydrogel with a denser cross‐linking network and increased cohesion. Owing to the hydrophobic segment in PLA, the in situ formed hydrogel can repel residual interfacial water to form tight contact with the tissue. Simultaneously, the functional groups in PGL can establish both covalent and non‐covalent bonds with the functional groups in the tissue, inducing robust adhesion between them (**Figure** **3**a). Therefore, by injecting PGL ethanol gel onto wet objects, such as rat liver, lung, heart, kidney, spleen, muscle, glass bottle, and metal, these objects can adhere to the glass plate via in situ formed PGL hydrogel and hold their own weights (Figure 3b).

To study the adhesion mechanism of the PGL ethanol gel‐derived hydrogel, we first measured the contact angle of water on various substrates, including a polyacrylamide (PAAm) hydrogel, glass plate, PGL hydrogel, and Teflon plate. The contact angles of the highly hydrophilic PAAm hydrogel, hydrophilic glass plate, PGL hydrogel, and hydrophobic Teflon plate were 18.9 ± 2.3°, 40.5 ± 4.5°, 65.6 ± 6.7°, and 98.3 ± 5.1°, respectively, which indicate the slight hydrophobicity of the PGL hydrogel (Figure 3c). Then, by depositing the PGL ethanol gel and PAAm hydrogel into a culture dish containing a rhodamine aqueous solution, the PGL ethanol gel can undergo solvent exchange and repel water synchronously. Thus, there was no residual rhodamine aqueous solution between the PGL ethanol gel and the dish. In contrast, the PAAm hydrogel could not repel water, leading to the presence of the rhodamine aqueous solution between the hydrogel and dish (Figure 3d; Video S2, Supporting Information). Next, we deposited PGL ethanol gel onto a wet porcine liver and a piece of porcine skin and observed the cross‐section of the samples using a fluorescence microscope and scanning electron microscope (SEM). As shown in Figure 3e and Figure S2 (Supporting Information), there were no gaps between the PGL hydrogel and the porcine liver/skin. These results demonstrate that the in situ formed PGL hydrogel can effectively repel interfacial water to form tight contact with the tissue for robust adhesion.

Next, we quantitatively evaluated the adhesive properties of the PGL ethanol gel‐derived hydrogel through a lap shear test, 180° peeling test, and tensile test (Figure 3f–h). The shear strength of the PGL ethanol gel‐derived hydrogel on porcine skin, heart, liver, and lung were 35.7 ± 4.0, 40.6 ± 4.1 kPa, 49.0 ± 9.4, and 40.8 ± 0.5 kPa, respectively, which were higher than those of commercial injectable fibrin gel (13.6 ± 2.1, 14.9 ± 5.2, 13.0 ± 2.9, and 14.3 ± 4.4 kPa, respectively). Furthermore, the adhesion toughness of the PGL ethanol gel‐derived hydrogel on porcine skin, heart, liver, and lung was determined to be 110.6 ± 31.6, 119.8 ± 12.7, 118.6 ± 21.9, and 130.3 ± 26.0 J m^−2^, respectively, exceeding those of commercial injectable fibrin gel (39.3 ± 12.6, 47.6 ± 6.9, 48.9 ± 13.1, and 46.4 ± 7.8 J m^−2^_,_ respectively). Besides, the adhesion tensile strength of the PGL ethanol gel‐derived hydrogel on porcine skin, heart, liver and lung was measured at 59.7 ± 7.0, 64.9 ± 14.2, 78.1 ± 14.6, and 65.0 ± 16.3 kPa, respectively, surpassing those of commercial injectable fibrin gel (26.8 ± 5.4, 23.9 ± 4.1, 27.1 ± 6.6, and 23.2 ± 10.1 kPa, respectively). In addition, we tested the change in the shear strength of the PGL ethanol gel‐derived hydrogel after immersing samples in an aqueous solution at different times. With the increase of immersing time, the solvent exchange ratio and cohesion of the PGL hydrogel increased obviously, resulting in increased shear strength (Figure 2h,i; Figure S3, Supporting Information). These results provide further evidence of the robust bioadhesion of the PGL ethanol gel‐derived hydrogel on wet tissues.

### 2.3 In Vitro and In Vivo Biocompatibility of the PGL Ethanol Gel

**Figure 4:**
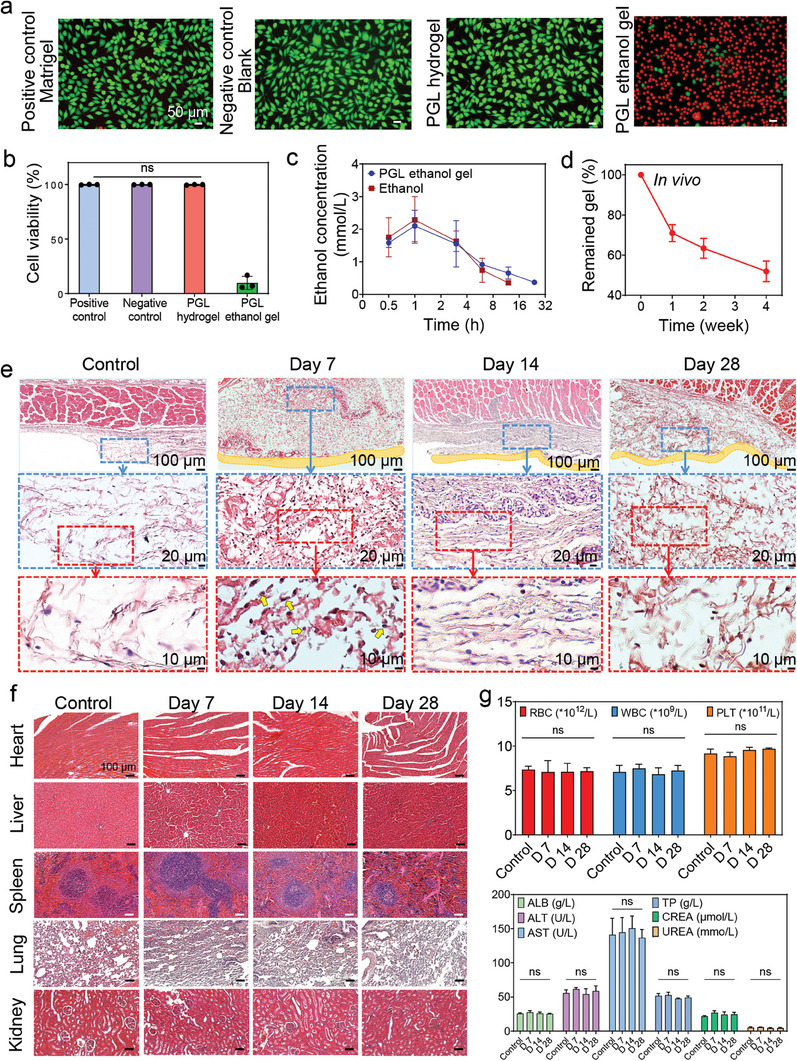
Biocompatibility and biodegradability of the PGL ethanol gel. a,b) Live/Dead staining (a) and cell viabilities (b) of L929 cells after being co‐incubated with different samples including Matrigel, Blank, PGL hydrogel, and PGL ethanol gel for 24 h (*n* = 3). c) Changes of ethanol concentration in the blood of rats after injection of the PGL ethanol gel (400 µL) and pure ethanol (400 µL) into their dorsal subcutaneous. d) In vivo degradation of the PGL ethanol gel. e) H&E staining images of the control tissue and tissue around the PGL ethanol gel‐derived hydrogel (original magnification × 10, × 40, and × 100). The yellow outline indicates the hydrogel and the yellow arrows indicate the neutrophils. f) Representative H&E staining images of heart, liver, spleen, lung, and kidney in control rats and experimental rats receiving PGL ethanol gel implantation. g) Blood routine tests (above) and serum biochemical tests (bottom) of control rats and experimental rats receiving PGL ethanol gel implantation. Rats, *n* = 3. RBC: red blood cell, WBC: white blood cell, PLT: platelets, ALB: albumin, ALT: alanine aminotransferase, AST: aspartate aminotransferase, TP: total protein, CREA: creatinine, UREA: blood urea. Statistical significance was calculated by One‐way analysis of variance (ANOVA); ns: not significant. Data are shown as the mean ± SD.

To evaluate the biocompatibility of the PGL ethanol gel and its derived hydrogel, they were incubated with L929 cells. After 24 h, the cell viability of the PGL hydrogel was higher than 99%, with no statistical difference compared to the positive control group and the negative control group. L929 cells, stained green with calcein/acetoxymethyl ester (calcein/AM), revealed a spindle‐like morphology in different groups (**Figure** **4**a,b). However, the cell viability of the PGL ethanol gel was lower than 10% because of the prolonged high levels of alcohol. Moreover, we injected the PGL ethanol gel (400 µL) and pure ethanol (400 µL) into the dorsal subcutaneous of the rats to test the ethanol concentration in their blood. After injection, the ethanol concentration in both groups increased over time and reached its peak within 1 h. Then, the ethanol concentration decreased over time. 24 h later, the ethanol concentration in the ethanol group was lower than the detection range, and there was little ethanol (0.37 ± 0.07 mmol L^−1^) in the PGL ethanol gel group (Figure 4c).

Next, the PGL ethanol gels were directly injected into the dorsal subcutaneous tissues of rats to evaluate their biocompatibility. After injection, blood was collected from the rats at 7, 14, and 28 d for routine blood and biochemical analyses. As shown in Figure 4g, there were no significant differences observed in routine blood and biochemical analysis tests (such as red blood cells, white blood cells, platelets, albumin, alanine aminotransferase, aspartate aminotransferase, total protein, creatinine, and blood urea) between the control and experimental rats that received PGL ethanol gel injection. This suggests that the PGL ethanol gel‐derived hydrogel did not cause systemic inflammatory response syndrome. The tissues around the PGL ethanol gel‐derived hydrogels were observed by hematoxylin and eosin (H&E) staining. On day 7, the PGL hydrogel (yellow dotted outline) induced a mild acute inflammatory infiltration containing neutrophils (yellow arrow). The number of inflammatory cells significantly reduced on days 14 and 28, indicating a reduced local inflammatory response (Figure 4e). In addition, H&E‐stained images of other vital organs (such as the heart, liver, spleen, lung, and kidney) exhibited no substantial differences between the experimental and control groups (Figure 4f). Besides, the PGL ethanol gel implanted in rats can degrade, with a degradation rate of ≈48.1% after 28 d (Figure 4d). And the clearance of the PGL hydrogels can be explained as follows: most of LA is mainly excreted in the urine via the kidneys in vivo, and small parts enter the cells to participate in energy metabolism^[^ ^16^ ^]^. And GA is mainly metabolized by the liver and kidneys^[^ ^17^ ^]^. In general, these results demonstrated the excellent biocompatibility and biodegradation of the PGL hydrogel in vitro and in vivo.

### 2.4 In Vitro Antitumor Efficacy of the PGL/DOX Ethanol Gel

**Figure 5:**
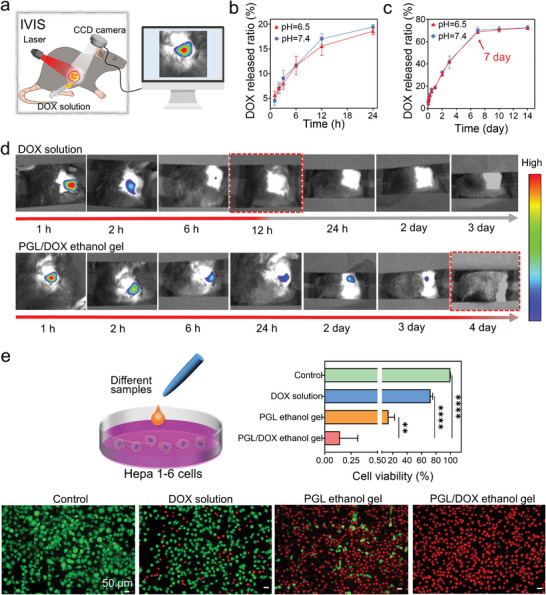
In vitro antitumor efficacy of the PGL/DOX ethanol gel. a) Schematic illustration for IVIS. b,c) In vitro DOX released ratio of the PGL/DOX ethanol gel in PBS with different pH. d) IVIS result for evaluating the remained behavior of free DOX solution and PGL/DOX ethanol gel after being injected into the tumors. e) The ability of the various samples including PGL/DOX ethanol gel, PGL ethanol gel, DOX solution, and PBS to kill Hepa1‐6 cells in vitro. *n* = 3. Statistical significance was calculated by One‐way ANOVA. ^**^ *p* < 0.01. ^****^ *p* < 0.0001. Data are shown as the mean ± SD.

To enhance the antitumor efficacy of the PGL ethanol gel, we introduced DOX into it to prepare a PGL/DOX ethanol gel. First, in vitro DOX release ratios in phosphate‐buffered saline (PBS) at different pH values (neutral and weakly acidic) were tested. Over the first 24 h, PGL hydrogels released 19.4 ± 0.5% and 18.5 ± 0.7% in neutral and weak acid SPF. Afterward, the DOX release ratios gradually increased, reaching 72.4 ± 2.8% and 70.4 ± 1.3%, respectively, at day 7 (**Figure** **5**b,c). This result indicated that the PGL/DOX hydrogel can achieve slow and sustained DOX release in vitro. Afterward, DOX solution and PGL/DOX (0.04 mL) were injected into the subcutaneous Hepa 1–6 tumor of the mice, and the DOX in vivo retention behavior was observed using image visualization and infrared spectroscopy (IVIS) (Figure 5a). As shown in Figure 5d, DOX in the PGL hydrogel was retained for 3 d in the tumor, while free DOX was rapidly eliminated within 12 h. These results imply that the PGL hydrogel serves as an ideal system for the sustained delivery of DOX in tumors.

Next, we evaluated the in vitro cytotoxicity of various samples to kill Hepa1‐6 cells, including DOX solution, PGL ethanol gel, and PGL/DOX ethanol gel. In the free DOX group, the cell viability was 72.5 ± 2.9%. However, when 0.02 mL PGL ethanol gel was added to the culture dish, the Hepa1‐6 cells shrunk rapidly due to the ethanol‐induced dehydration, resulting in a cell viability of 14.4 ± 8.1%. Subsequently, the PGL/DOX hydrogel released DOX to further kill Hepa1‐6 cells, resulting in the lowest cell viability (0.1 ± 0.17%). Therefore, these results demonstrate the effective in vitro killing of tumor cells by the PGL/DOX ethanol gel.

### 2.5 In Vivo Antitumor Efficacy of the PGL/DOX Ethanol Gel

**Figure 6:**
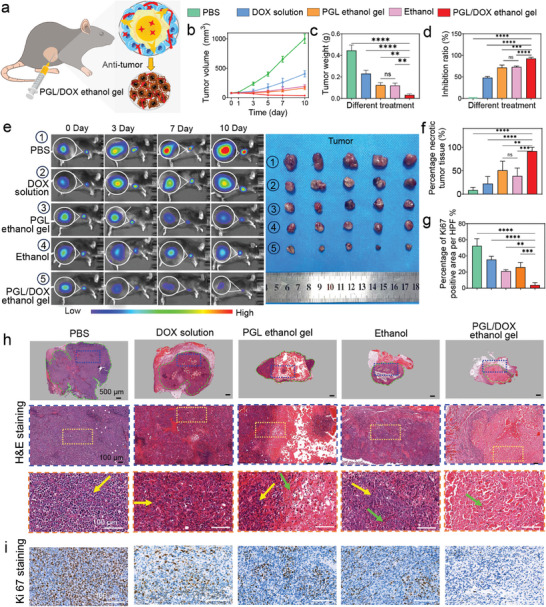
In vivo antitumor efficacy of the PGL/DOX ethanol gel. a) Schematic illustration for treating C57BL/6 mice bearing subcutaneous Hepa 1–6 tumors by in situ injecting PLG/DOX ethanol gel. b–d) Change of tumor volume (b), tumor weight (c), and inhibition ratio (d) of the mice treated with various samples including PBS, DOX solution, PGL ethanol gel, ethanol, and PGL/DOX ethanol gel. e) IVIS (left) and tumors (right) images for groups treated with various samples. f,g) Percentage of necrotic tumor tissue (f) and percentage of Ki67 positive area at high power field (HPF) in various groups. h,i) H&E staining (h) and Ki67 staining (i) of the tumors in various groups. *n* = 5. Statistical significance was calculated by One‐way ANOVA; ns: not significant. ^*^ *p* < 0.05. ^**^ *p* < 0.01. ^***^ *p* < 0.001. ^****^ *p* < 0.0001. Data are shown as the mean ± SD.

To evaluate in vivo antitumor efficacy of the PGL/DOX gel, experiments were performed on C57BL/6 mice bearing subcutaneous Hepa 1–6 tumors (**Figure** **6**a). The mice were randomly divided into five groups and were injected (into the tumors) with PBS, DOX solution, PGL ethanol gel, ethanol, and PGL/DOX ethanol gel, respectively. The tumor volumes were monitored for 10 d. The free DOX solution group displayed limited antitumor efficacy as free DOX in the tumor was rapidly eliminated. In contrast, the ethanol and PGL ethanol gel groups demonstrated significant antitumor efficacy, indicating that ethanol can effectively kill tumor cells upon contact with the tumor tissues. Furthermore, the PGL/DOX ethanol gel revealed the best antitumor efficacy, as the in situ formed PGL/DOX hydrogel could sustainably release DOX to further enhance antitumor efficacy (Figure 6b,e). To further evaluate antitumor efficacy, we removed the tumors, weighed the weight of the tumor, and calculated the inhibition ratio (Figure 6c,d). The tumors in the PGL/DOX ethanol gel group had the smallest tumor volumes and weights and the maximum inhibition ratio (86.4 ± 6.8%), further underscoring the excellent antitumor efficacy of the PGL/DOX ethanol gel. In addition, the body weight of all mice displayed no obvious variation, and there was negligible damage to the main organs based on H&E staining images (Figures S4 and S5, Supporting Information), indicating the negligible side toxicity of these samples.

Next, H&E‐stained images were used to further evaluate antitumor efficacy. As shown in Figure 6h, there were a large number of tumor cells (yellow arrow) in the PBS and DOX groups. In the ethanol and PGL ethanol gel groups, there was evident nuclear condensation or dissolution (green arrow). However, there were still many residual tumor cells in the tissue that had not been exposed to ethanol. In contrast, the PGL/DOX ethanol gel induced more nuclear condensation or dissolution, and fewer residual tumor cells. Quantitative evaluation revealed that the proportion of tumor necrosis in PBS, DOX solution, PGL ethanol gel, ethanol, and PGL/DOX ethanol gel were 7.3 ± 5.9%, 23.8 ± 13.5%, 52.7 ± 17.5%, 41.1 ± 15.4%, and 85.8 ± 7.0%, respectively (Figure 6f,h). We further evaluated antitumor efficacy by immunohistochemical staining against Ki67 (a nuclear marker for proliferating cells). Ki67 staining indicated that the percentage of the Ki67‐positive cells in PBS, DOX solution, PGL ethanol gel, ethanol, and PGL/DOX ethanol gel were 56.7 ± 12.4%, 39.4 ± 9.5%, 23.2 ± 4.7%, 27.6 ± 6.2%, and 4.6 ± 2.9%, respectively, highlighting the best antitumor efficacy of the PGL/DOX ethanol gel. Taken together, after injecting the PGL/DOX ethanol gel into the subcutaneous Hepa 1–6 tumor, ethanol first killed the tumor cells and formed a hydrogel in situ through solvent exchange. The hydrogel sustainably released DOX to kill tumor cells, resulting in excellent antitumor efficacy.

## 3 Conclusion

In conclusion, we have introduced a highly promising solvent‐exchange strategy for preparing adhesive hydrogels that can be used to treat solid tumors via synchronous ethanol ablation and local chemotherapy. A PGL ethanol gel was obtained by the one‐pot reaction of GA and LA in a good solvent (ethanol). By depositing the PGL ethanol gel onto wet tissues, it can immediately undergo solvent exchange to form a hydrogel in situ due to the formation of intra‐ and inter‐polymer hydrogen bonding and hydrophobic aggregation. The in situ formed PGL hydrogel can repel residual interfacial water, establishing tight contact with the tissues. The functional groups between the hydrogels and tissues can form covalent and noncovalent bonds with the functional groups in the tissue, resulting in robust adhesion between them. The obtained PGL hydrogels exhibited excellent biocompatibility and biodegradability. Furthermore, this PGL ethanol gel can effectively load the antitumor drug DOX and release it locally and sustainably. In addition, by combining ethanol ablation and local chemotherapy, the PGL/DOX ethanol gel exhibited effective antitumor efficacy in vitro and in vivo. Moreover, with the increased tumor volume (from mouse to human‐sized tumors), the penetration distance from the injection point to the periphery of the tumor increases, which leads to decreased effective penetration and therapeutic effect. Thus, to improve the therapeutic effect for larger tumors, the dosage of ethanol gel or DOX can be increased according to practical situations. This solvent‐exchange‐induced hydrogel offers the following advantages: robust wet bioadhesion, drug loading, and sustained release, good biocompatibility and biodegradability, easy preparation and usage, and cost‐effectiveness. These qualities make it an ideal bioadhesive with promising potential for diverse biomedical applications, such as wearable devices, wound dressings, and drug delivery.

## 4 Experimental Section

### Materials

LA and GA were purchased from Aladdin (China). DOX was procured from Macklin (Beijing, China). Absolute ethanol was sourced from the Guangzhou Chemical Reagent Factory (Guangzhou, China). Dulbecco's modified Eagle's medium (DMEM) and PBS were purchased from Gibco (USA).

### PGL Ethanol Gel and Hydrogel Preparation

Dissolving GA and LA in absolute ethanol and heating the solution at 70 °C for 4 h to obtain the PGL ethanol gel, which was injected into water or body fluid to form the PGL hydrogel.

### Characterizations

Raman spectra of the LA powder and dried PGL hydrogel were recorded using a Raman spectrometer (Renishaw inVia Qontor, USA) at an excitation wavelength of 785 nm. FTIR (Thermo Scientific Nicolet iS10, USA) was used to analyze LA, GA, PGL ethanol gel, and dried PGL hydrogel. A Phenom Pure Desktop scanning electron microscope (Thermo Scientific, USA) was used to observe the interface between the porcine tissues and adhered PGL ethanol gel‐derived hydrogels.

### Rheology Tests

The rheological properties of the PGL ethanol gels and PGL hydrogels were tested using an Anton Paar MCR 92 rheometer. The dynamic strain sweep measurements of PGL ethanol gel were carried out at 1–1000% strain and fixed angular frequency of 10 rad s^−1^. Time–sweep measurements of PGL hydrogel were performed at a fixed strain of 1% and a constant angular frequency (10 rad s^−1^). The step‐strain rheological experiment changed strain from 1% to 1000% and back from 1000% to 1%. Temperature = 37 ± 0.2 °C.

### Solvent Exchange Ratio In Vitro

One milliliter of the PGL ethanol gel was injected into 10 mL of water. Afterward, the solution was replaced and collected at pre‐determined times to test the ethanol concentration using an ethanol colorimetric assay kit. (Elabscience, E‐BC‐K891‐M)

### Mechanical Tests

To evaluate the adhesive performance of the PGL ethanol gel‐derived hydrogel quantitatively, we measured the shear strength using a lap shear test, interfacial toughness was assessed via a 180° peeling test, and tensile strength was determined with a tensile test. For these tests, porcine tissues, including porcine skin, heart, liver, and lungs, were cut into strips (length: 40 mm; width: 10 mm). Two pieces of identical tissues were stuck by the PGL ethanol gel‐derived hydrogels at different times (0.5, 1, 3, 6h) underwater without any additional treatment. These tissues adhered to the PGL ethanol gel‐derived hydrogels. An electronic universal testing machine (ZQ‐990L, China) was employed to store samples at a crosshead speed of 10 mm min^−1^. The formulas used for the calculations are as follows: Shear strength = *F*_max_/*WL*, Interfacial toughness = *F*_plateau_/*W*, Tensile strength = *F*_tensile_/*WL* (*F*_max_: maximum force of the lap shear test; *F*_plateau_: plateau force of the 180° peeling test, *F*_tensile_: maximum force of the tensile test; and *W* and *L* denote the width and length of the adhesive area, respectively). Three standardized samples were tested in each experiment to ensure the reliability of the results.

### Contact Angle Test

The contact angles of water on various samples, including a PAAm hydrogel, glass plate, PGL hydrogel, and Teflon plate were determined at room temperature using a surface contact angle meter (POWEREACH, China). Dropping 20 µL of deionized water onto the sample surface and recording at least five distinct points for each sample. The average contact angle was calculated from five measurements per sample using JC2000D software.

### In Vitro Biocompatibility

The live/dead staining method was used to evaluate the biocompatibility of the PGL ethanol and its derived hydrogel. NCTC clone 929 (L929) cells were added to 24‐well plates at 20 000 cells per well and cultured at 37 °C for 24 h. Subsequently, 20 mg of PGL ethanol gel, the hydrogel, or Matrigel was added to the wells and co‐cultured with the cells for 24h, respectively. Afterward, the culture medium was removed, and the cells were washed twice with PBS. Then, 250 µL of staining dye (Calcein‐AM/propidium iodide dye) was added. Finally, the cells were observed under a fluorescence microscope.

### In Vivo Biocompatibility and Degradation Tests

Sprague–Dawley rats (Guangdong Viton Lihua Laboratory Animal Technology Co.) were used to test the in vivo biocompatibility of the PGL ethanol gels and the degradation of the PGL hydrogels. All experimental rats complied with the animal ethics guidelines of the Animal Ethics Committee of the Fifth Affiliated Hospital of Sun Yat‐sen University (00363). The PGL ethanol gels (0.4ml) were injected into the subcutaneous tissue of the rat back. On days 7, 14, and 28, the rats were euthanized. The hydrogels were removed and weighed to calculate their degradation ratios. The degradation ratio of each sample was calculated according to the following formula: degradation ratio (%) = (*m*_0_ – *m*_1_)/ *m*_0_ × 100% (*m*_1_ and *m*_0_ represent the final weights and original weights of the dried gels, respectively). The tissues surrounding the hydrogels were fixed in phosphate‐buffered formalin (Biosharp, 1810083) for 24 h, dehydrated, and embedded in paraffin.

### The Rat's Blood Alcohol Level Tests

Rats were divided into two groups and injected subcutaneously with 0.4 mL ethanol and the PGL ethanol gel, respectively. After the rats’ blood collection at pre‐determined times, the concentration was tested using an ethanol colorimetric assay kit. (Elabscience, E‐BC‐K891‐M)

### In Vitro Antitumor Tests

Hepa 1–6 cells from mice were added to 24‐well plates at 20 000 cells per well and were cultured at 37 °C for 24 h. Afterward, PBS, 0.02 mL DOX solution (0.25 mg mL^−1^), PGL ethanol gel (0.02 mL), and 0.02 mL PGL/DOX ethanol gel containing 0.005 mg DOX were added to the well, respectively. After co‐culturing for 12 h, the cells were stained with calcein‐AM/propidium iodide dye and observed under a fluorescence microscope.

### Drug Release Tests

DOX was capable of autofluorescence, and the relationship between DOX and fluorescence intensity was proportional to the concentration range. First, different concentrations of DOX hydrochloride solutions were prepared, the absorbance was measured, and a standard curve was plotted using the fluorescence module of an enzyme labeling instrument (Biotek, USA, excitation wavelength: 530 nm, emission wavelength: 590 nm). DOX (1 mg) was encapsulated in 1 mL of PGL ethanol gel and left at room temperature for 24 h. After that, 500 µL of the PGL/DOX ethanol gel was injected into 2 mL of PBS at pH 6.5 and 7.4. One microliter of the supernatant was collected and diluted at different time points, the absorbance was measured, and the release rate was calculated.

### In Vivo Drug Retention Test

Injecting 0.04 mL DOX solution (1 mg mL^−1^) and 0.04 mL PGL/DOX ethanol gel (1 mg mL^−1^) into the Hepa 1–6 tumor‐bearing mice. At a pre‐set time, fluorescence images of the mice were collected to evaluate in vivo DOX release. All experimental mice complied with the animal ethics guidelines of the Animal Ethics Committee of the Fifth Affiliated Hospital of Sun Yat‐sen University (00363). All experimental mice were provided by Zhuhai Bestest Biotechnology Co., Ltd and acclimated to the environment for 1 week. The in vivo imaging system was PerkinElmer IVIS Lumina III; Imaging mode: Fluorescence; Excitation filter:520 nm; emission filter:570 nm.

### In Vivo Inhibition Efficiency Against Tumor Test

To establish the Hepa 1–6 tumor model in mice, 100 µL PBS containing 2 × 10^6^ Hepa 1–6 cells were injected into the right axilla of the C57BL/6 mice. When the tumor volume reached ≈70.0 mm^3^, the mice were randomly divided into five groups. Then, 40 µL PBS, 40 µL DOX solution (1 mg mL^−1^), 40 µL PGL ethanol gel, 40 µL absolute ethanol, or 40 µL PGL/DOX ethanol gel (1 mg mL^−1^) were directly injected into the tumor of these mice. The tumor volume and body weight of the mice were measured at pre‐determined times. Tumor volume was calculated according to the following formula: volume = width^2^ × length/2.^[^ ^18^ ^]^ In addition, the growth of the tumor was recorded by the in vivo imaging system (Lumina III) on days 0, 3, 7, and 10. Moreover, the tumors were removed and fixed in phosphate‐buffered formalin (Biosharp, 1810083), dehydrated, and embedded in paraffin.

### Hematoxylin‐Eosin (H&E) Staining

The embedded samples were cut into slides with a 5 µm thickness. The slides were heated at 60 °C for 10 min, dewaxed with xylene, dehydrated using an alcohol gradient, and rehydrated in deionized water. The slides were then stained with hematoxylin and eosin according to established protocols.

### Immunohistochemistry (IHC) Staining

Paraffin slices of tumors were dewaxed, dehydrated, and incubated with primary antibodies (Ki67, CST) and secondary antibodies (IgG, Abcam). After PBS washing, the slices were stained with 100 µL 3,3′‐diaminobenzidine (DAB) and counterstained with hematoxylin.

### Statistical Analysis

Statistical computations were performed using GraphPad Prism version 9.5. All data were presented as mean ± SD. All experimental data were compared using Student's t‐test and one‐way ANOVA, followed by Tukey's post hoc analysis to determine the level of statistical significance between two or multiple groups. Significant differences between groups were indicated by ^*^ *p* < 0.05, ^**^ *p* < 0.01, ^***^ *p* < 0.001, and ^****^ *p* < 0.0001.

## Conflict of Interest

X.P. and Y.C. are named as inventors on a patent application (ZL 202311651882.3) that covers the design and applications of the PGL gel. The authors declare that they have no other competing interests.

## Author Contributions

Y.C. and M.Y. contributed equally to this work. X.P. conceived and designed the experiments. Y.C. and Y.M. performed the material preparations, characterization, and in vitro experiments. Y.C., M.L., C.L., Y.S., M.Y., W. Z., and W.Z. conducted the experiments of animal experiments. Y.C. and X.P. wrote the paper. All authors commented on the manuscript.

## Supporting information

Supporting Information

Supplemental Video 1

Supplemental Video 2

Supplemental Table 1

## Data Availability

The data that support the findings of this study are available from the corresponding author upon reasonable request.
