# Supplementary material for: A Solvent Exchange Induced Robust Wet Adhesive Hydrogels to Treat Solid Tumor Through Synchronous Ethanol Ablation and Chemotherapy
Source: Adv Sci (Weinh). 2024 Apr 6;11(24):2309760. doi: 10.1002/advs.202309760 (PMC11200021; doi:10.1002/advs.202309760)
Supplement: Supplementary file 1 — Supporting Information [file ADVS-11-2309760-s001.pdf]

ADVANCED SCIENCE
Open Access

Supporting Information

for *Adv. Sci.*, DOI 10.1002/advs.202309760

A Solvent Exchange Induced Robust Wet Adhesive Hydrogels to Treat Solid Tumor Through Synchronous Ethanol Ablation and Chemotherapy

*Yanlv Chen, Meng Yu, Menghui Liu, Yang Sun, Chengxian Ling, Mingyu Yu, Wenwen Zhang, Wenkai Zhang and Xin Peng**

Supporting Information

A Solvent Exchange Induced Robust Wet Adhesive Hydrogels to Treat Solid Tumor through Synchronous Ethanol Ablation and Chemotherapy

*Yanlv Chen†, Meng Yu†, Menghui Liu, Yang Sun, Chengxian Ling, Mingyu Yu, Wenwen Zhang, Wenkai Zhang, Xin Peng**

Supporting Figures

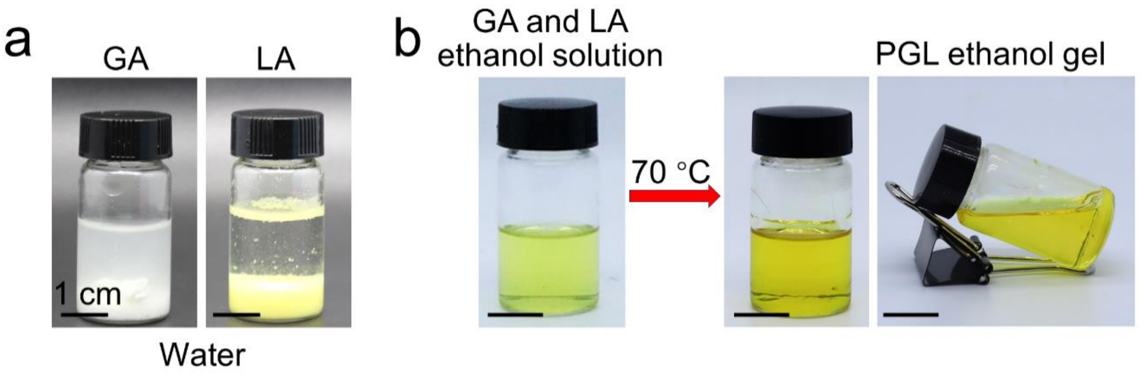

Figure S1. (a) Dissolution of GA and LA in water. (b) GA/LA ethanol solution and PGL ethanol gel.

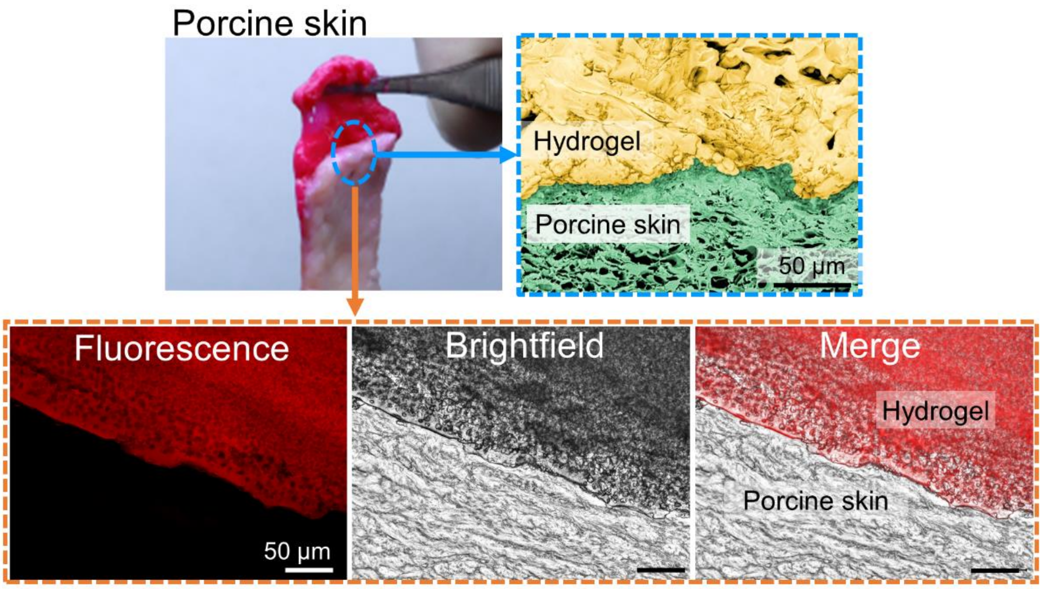

Figure S2. Microscopic and SEM photos of the cross section of the interface of the PGL hydrogel and porcine skin.

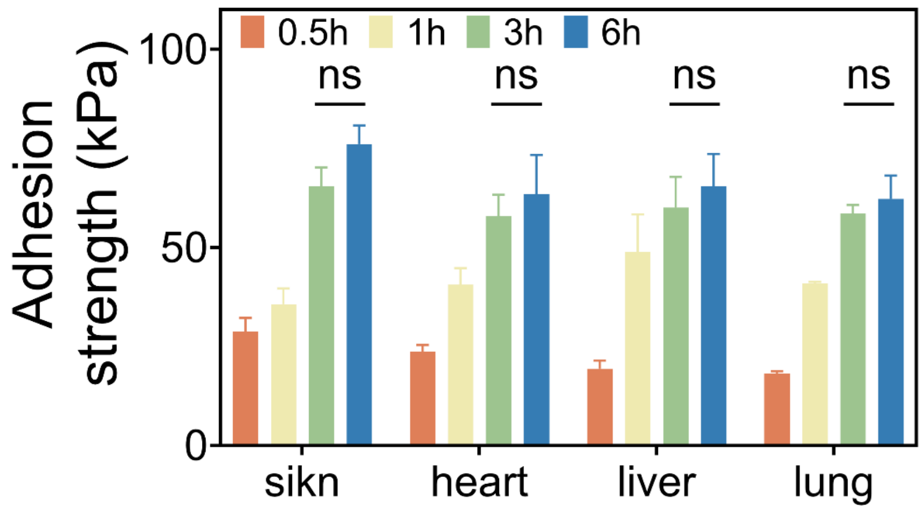

Figure S3. The adhesive performances of the PGL ethanol gel-derived hydrogel by testing their shear strength.

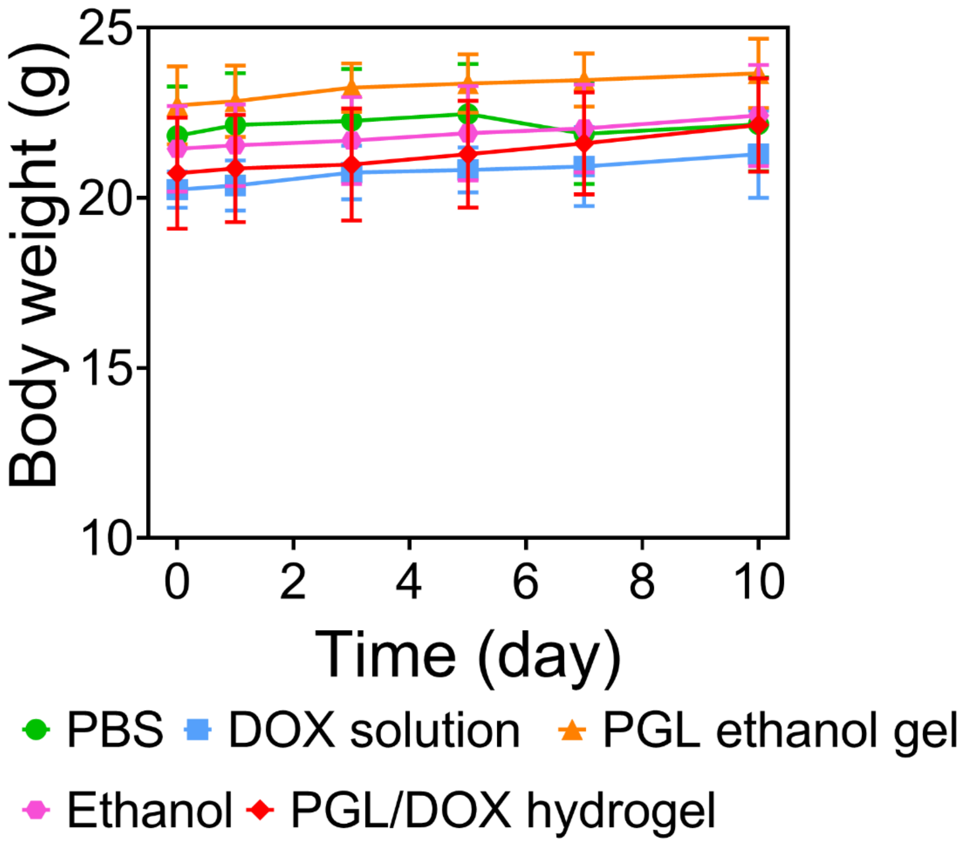

Figure S4. Change of the weight of the C57BL/6 mice bearing subcutaneous Hepa 1-6 tumors treated with various samples including PBS, DOX solution, PGL ethanol gel, ethanol and PGL/DOX ethanol gel. *n* = 5. Data are shown as the mean ± SD.

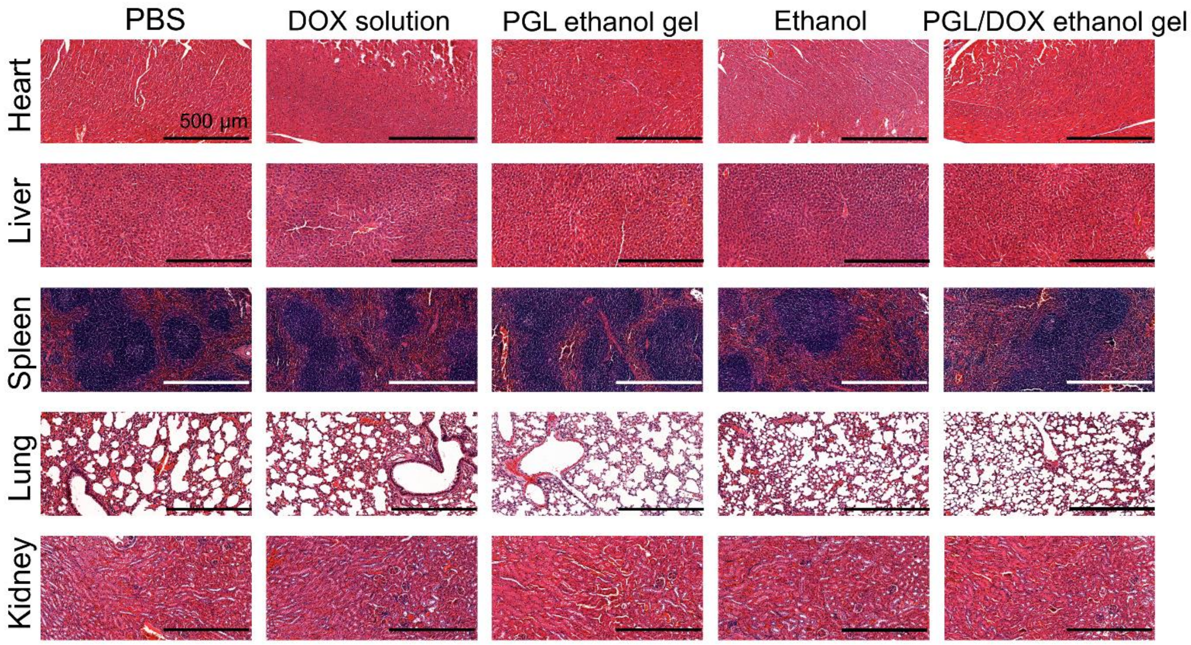

Figure S5. Representative H&E staining images of heart, liver, spleen, lung, and kidney in the C57BL/6 mice bearing subcutaneous Hepa 1-6 tumors treated with various samples including PBS, DOX solution, PGL ethanol gel, ethanol and PGL/DOX ethanol gel.
